# The synergy and mode of action of quercetin plus amoxicillin against amoxicillin-resistant *Staphylococcus epidermidis*

**DOI:** 10.1186/s40360-016-0083-8

**Published:** 2016-08-04

**Authors:** Supatcharee Siriwong, Yothin Teethaisong, Kanjana Thumanu, Benjawan Dunkhunthod, Griangsak Eumkeb

**Affiliations:** 1 School of Pharmacology, Institute of Science, Suranaree University of Technology, 111 University Avenue, Suranaree Subdistrict, Muang District, Nakhonratchasima, 30000 Thailand; 2 Synchrotron Light Research Institute (Public Organization), Suranaree Subdistrict, Muang District, Nakhonratchasima, 30000 Thailand

**Keywords:** Quercetin, Kaempferol, Amoxicillin, Penicillin, Amoxicillin-resistant *Staphylococcus epidermidis*, Synergistic activity, Mechanism of action

## Abstract

**Background:**

*Staphylococcus epidermidis* is one of the most multiple resistances to antibiotics in the recent years. Therefore, practically-prescribed antibiotics in the treatment of these strains are not effective. Plant-derived antibacterial is one of the most interesting sources of new therapeutics. The present study was to investigate antibacterial, synergy and modes of action of quercetin and amoxicillin against amoxicillin-resistant *Staphylococcus epidermidis* (ARSE).

**Methods:**

The MICs, checkerboard assay, viability curves, cytoplasmic membrane (CM) permeability, enzyme assay, transmission electron microscopy, confocal microscopy and FT-IR microspectroscopy measurement was performed.

**Results:**

The MICs of amoxicillin, penicillin, quercetin and kaempferol against all ARSE strains were 16, 200, 256-384 and >1024 μg/mL respectively. Synergistic effects were exhibited on amoxicillin plus quercetin and penicillin plus kaempferol against these strains at FIC index 0.50 and <0.38 respectively. The synergistic activity of quercetin plus amoxicillin was confirmed by the viable count. This combination increased CM permeability, caused marked morphological, peptidoglycan and cytoplasmic membrane damage, increased protein amide I and II, but decreased fatty acid in bacterial cells. The quercetin had an inhibitory activity against β-lactamase.

**Conclusions:**

So, these findings are the first report that quercetin has the synergistic effect with amoxicillin against ARSE via four modes of actions, inhibit peptidoglycan synthesis and β-lactamases activity, increase CM permeability and protein amide I and II but decrease fatty acid in bacterial cells. Of course, this flavonol has the dominant potential to develop a brand-new collateral phytochemical agent plus amoxicillin to treat ARSE. Future work should focus on the bioavailability, efficacy and toxicity in animal and human studies, as well as, the synergistic effect on blood and tissue should be evaluated and achieved.

## Background

**Fig. 1:**
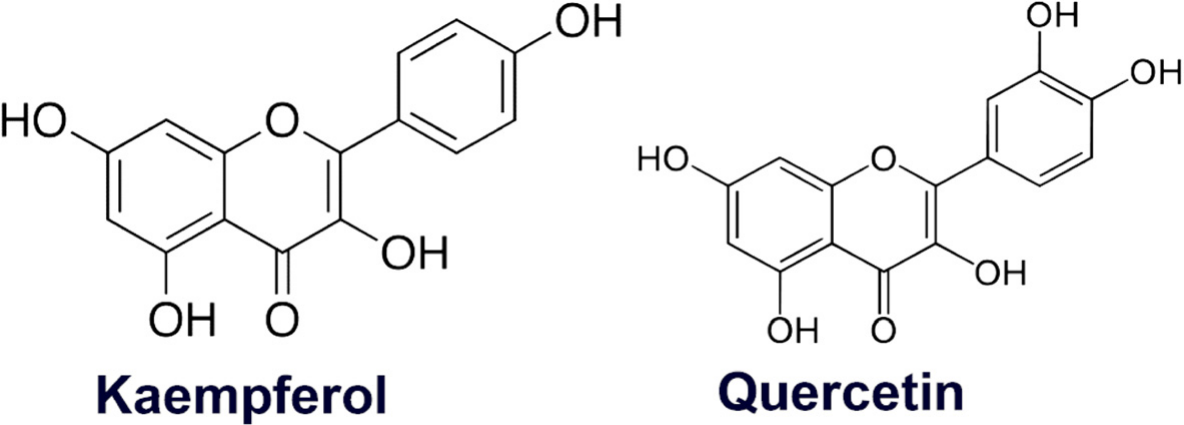
The chemical structure of Kaempferol and Quercetin

In the recent years, the incidence of multidrug resistance in pathogenic and opportunistic bacteria has been increasingly documented. These bacteria pose life-threatening risks to the hospitalized patients and their caregivers [1]. *Staphylococci* are one of the most numerous resistances to many new and commonly prescribed antibiotics in the current year as a consequence of the selective pressure produced by therapeutic misuse of antibiotics and abuse [2]. Both strains of *Staphylococcus aureus* and *Staphylococcus epidermidis* have accumulated multiple resistance determinants [3]. One mode of penicillin-resistant action of bacteria is by producing β-lactamase to destroy penicillins [4]. Consequently, practically-prescribed antibiotics in the treatment of these strains are not effective. Antibacterial agents available for the treatment of *S. epidermidis* infection exhibit toxicity and their use are frequently associated with high cost and unwanted side-effects. Thus, searching and development of novel antibacterial compounds and new strategies that can reverse the resistance to well-established therapeutic agents are urgently required. Plant-derived antibacterial is one of the most interesting sources of new therapeutics. Quercetin that has been found in onions, tomatoes, and honey, showed potent antibacterial activity against a wide spectrum pathogen responsible for hospital- and community-acquired by bacterial DNA gyrase and topoisomerase IV inhibition [5]. Quercetin and kaempferol, both are classified as flavonols (Fig. 1), showed to limit cancer cell growth by inducing apoptotic cell death [6] and antibacterial activity against *Escherichia coli*, *Pseudomonas aeruginosa*, *Staphylococcus aureus*, and *Enterococcus faecalis* [7]. Kaempferol, that has been isolated from tea, broccoli, delphinium, witch-hazel, grapefruit, cabbage, kale, beans, endive, leek, tomato, strawberries, grapes, Brussels’ sprouts, apples and other plant sources such as the indigo plant (*Polygonum tinctorium* Lour.), were effective against *H. pylori* in vivo [8]. The effect of chlorine-induced bacterial injury on spectral features was investigated by using Fourier transform infrared (FT-IR) absorbance spectroscopy [9]. Moreover, FT-IR spectroscopy was used to verify the deformation of macromolecules in the bacterial membrane, upon treatment with eugenol [10]. Also, the mode of action and bimolecular such as fatty acid, nucleic acid and protein in *E. coli* envelope after exposure to luteolin either alone or in combination with amoxicillin were clearly obtained using FT-IR [11]. So, these studies provide evidence that an effective tool for an early evaluation of the efficiency of the anti-bacterial effect of other used drugs can be FT-IR spectroscopy [12]. The purpose of this study was to investigate the synergistic activity and mode of action of quercetin, the flavonol, plus amoxicillin against amoxicillin-resistant *S. epidermidis* strains.

## Methods

### Materials and bacterial strains

This study was a cross-sectional observational study to investigate the susceptibility profile of amoxicillin on *Staphylococcus epidermidis* isolates. These strains were obtained during the period between January and September 2015 from clinical samples of urine, wound pus, blood, and sputum, of several infection types of 736 patients admitted to the 60 public hospitals across Thailand. The study included only one clinical isolate per patient. The bacterial species and antibacterial susceptibility testing were performed at the Department of Medical Sciences, Ministry of Public Health and the Suranaree University of Technology, Thailand respectively. The 90 % failure rate for amoxicillin used for the treatment of *S. epidermidis* associated with indwelling catheters, other implanted devices, otitis, sinusitis, and pharyngeal-tonsillitis of these patients have been found. The amoxicillin-resistant *Staphylococcus epidermidis* strains (DMST 5038, 5023, 5868, 4248) (ARSE) were assigned by the Department of Medical Sciences, Ministry of Public Health, Thailand. *Staphylococcus aureus* ATCC 29213 (*S. aureus*), used as positive control, were purchased from the American Type Culture Collection (ATCC). Quercetin (purity 98 %) and Kaempferol (purity 99 %) (Fig. 1) were purchased from the Indofine Chemical Company (New Jersey, USA). Amoxicillin, penicillin, β-lactamase type IV, dimethyl sulfoxide (DMSO), glutaraldehyde (Grade I, 25 % for EM), osmium tetroxide (4 % for EM), Spurr Low-Viscosity Embedding Kit and nisin (from *Lactococcus lactis*, 2.5 % balance sodium chloride and denatured milk solids) were obtained from Sigma (Sigma-Aldrich, UK). Mueller–Hinton agar (MHA) and Mueller–Hinton broth (MHB) was obtained from Oxoid (Basingstoke, UK).

### Bacterial suspension standard curves

Bacterial suspensions standard curve method was performed to determine known viable count following the method of Richards and Xing [13] with little modifications. Briefly, the ARSE cultures were incubated in Cation-adjusted Mueller-Hinton broth (CAMHB) at 35 °C for 20 h. The bacterial cells were pelleted by centrifugation at 3000 *xg* and were washed twice by 0.9 % NaCl to remove media. The cells were resuspended in sterile 0.9 % NaCl and diluted so that 5–6 spectrophotometer readings could be obtained over the absorbance range of approximately 0.05–0.25 at a wavelength of 500 nm. Viable counts for each absorbance reading were determined in triplicates.

### Minimum inhibitory concentration (MIC) determination

MIC determinations of amoxicillin, penicillin, nisin, quercetin and kaempferol against ARSE strains were performed following the method of Liu et al. [14]; Eumkeb et al. [11] and Clinical and Laboratory Standards Institute [15]. In brief, the inoculums of 0.25 mL of 5×10^6^ CFU/mL bacterial suspension (20 h culture) was added to triplicate tubes containing 2.25 mL CAMHB plus an antibacterial or flavonols to give approximately 5×10^5^ CFU/mL in each tube. The working solutions of each test agent were prepared using serial dilutions from 1024 μg/mL up to 1.0 μg/mL. MIC determination was accomplished after 20 h of incubation at 35 °C by observing turbidity. The lowest concentrate of each agent that prevented bacterial growth was considered to be the MIC. Tubes of CAMHB without test agent were used as the control.

### Checkerboard determination

Checkerboard assay to determine the synergistic activity of flavonols in combination with penicillins against penicillin-resistant *S. epidermidis* strains were executed following Eumkeb et al. [11] and Bonapace et al. [16] To sum up briefly, the 0.25 mL of 5 × 10^6^ CFU/mL bacterial suspensions were added to a series of 2.25 mL CAMHB plus 10 %, serial dilution of the flavonol plus antibacterial combinations to give 5×10^5^ CFU/mL. Tubes of the broth without antibacterial cell were used as the control. The cultures were incubated for 20 h at 35 °C. The tests were carried out in triplicate. MICs were determined for each antibacterial combination and the isobolograms were plotted. The interaction between the two agents was calculated by the fractional inhibitory concentration (FIC) index of the combination. The following formula was used for FIC index calculation: FIC of quercetin = MIC quercetin in combination/MIC of quercetin alone; FIC of amoxicillin = MIC of amoxicillin in the combination/MIC of amoxicillin alone; So, FIC index = FIC of quercetin + FIC of amoxicillin. When the FIC index of the combination is equal to or less than 0.5, the combination is defined as synergistic; when FIC index falls between 0.5 and 4.0, it indicates ‘no interaction’ between the agents, and a value above four-term antagonism between the two compounds [17]. *S. aureus* ATCC 29213 was used as positive control. The MICs and FIC index is presented as the median values obtained in duplicates from three independent experiments.

### Determination of viability curves

The killing curve determination was performed to confirm the synergistic activity of the combination following Richards and Xing [13]; Eumkeb et al. [11] and Clinical and Laboratory Standards Institute [15] methods with slight modifications. To summarize, after the FIC index was obtained, the MIC of each compound that gave synergism FIC index of the combination was chosen to investigate. The half-MICs value of amoxicillin and quercetin alone and the MICs of this combination that gave synergistic FIC index value were picked against *S. epidermidis* DMST 5038 (ARSE 5038) [18]. So that, the concentration of 8 μg/mL amoxicillin, 128 μg/mL quercetin, amoxicillin at 4 μg/mL plus quercetin at 64 μg/mL combinations and control (without amoxicillin or quercetin) was tested. The cultures were prepared on CAMHB for 20 h at 35 °C. Inocula of 2 mL of culture were added into 98 mL CAMHB and shaking at 100 r.p.m. at 37 °C for 4 h to give log phase. Bacterial cultures were adjusted in saline to give 5 × 10^6^ CFU/mL. Log phase of the cultures was added to CAMHB plus amoxicillin and quercetin either alone, or combined agents at concentrations mention above. The bacterial suspensions were incubated at 37 °C in the shaker water bath. Viable counts were determined after a contact time of 0, 0.5, 1, 2, 4, 6, 8 and 24 h. Subsequent dilution plating on MHA agar plates in triplicate and incubation at 35 °C for 20 h were allowed the counting of growing colonies [19].

### Cytoplasmic membrane permeability

The cytoplasmic membrane permeabilization was performed as previously described by Shen et al. [20] and Zhou et al. [21] with some modifications. This method was performed by measurement the release of UV-absorbing material concentrations using UV-VIS spectrophotometer. The ARSE 5038 strain was conducted in this experiment. In brief, this ARSE culture was prepared on CAMHB for 20 h at 35 °C. Inocula of 2.0 mL of culture were added into 98.0 mL CAMHB and shaking at 100 r.p.m. at 37 °C for 4 h to give log phase. Bacterial cultures were adjusted in saline to give 5 × 10^6^ CFU/mL. Log phase of the adjusted cultures 1.0 mL was added to 9.0 mL of 2.5 mmol/L sodium HEPES buffer (pH 7.0) supplemented with 100 mmol/L glucose plus 8 μg/mL amoxicillin, 128 μg/mL quercetin (½ MIC), and 3 μg/mL amoxicillin plus 48 μg/mL quercetin (¾ FIC) in each flask to give a final concentration at 5 × 10^5^ CFU/mL. The flasks of cell suspension without antibacterial cell were used as the negative control and with 128 μg/mL nisin (½ MIC) was used as positive control. The bacterial suspensions were incubated at 37 °C in the shaker water bath. The CM permeability was determined after a contact time of 0, 0.5, 1.0, 2.0, 3.0 and 4.0 h. After treatment, samples (1.0 mL) were taken every contact time and filtered through a sterile nitrate cellulose membrane (0.22 μm), and OD_260_ value of the supernatant was taken as a percentage of the extracellular UV-absorbing materials released by cells. All the measurements were done in triplicates in Varian Cary 1E UV/ VIS spectrophotometer [11].

### Enzyme assay

Many isolated cultures of *Staphylococcus epidermidis* strains produced beta-lactamase [22]. Bacteria have produced β-lactamase enzymes that can inactivate β-lactam antibiotics by hydrolyzing the peptide bond of the β-lactam ring rendering the antibiotic ineffective [23]. Quercetin was investigated to clarify whether its had inhibitory activity against this enzyme or not by the β-lactamase assay [24]. Enzyme activities were performed following the method as previously described by Richards et al. [25]. Briefly, high performance liquid chromatography (HPLC) was used to measure the stability of benzylpenicillin to β-lactamase in the presence of an enzyme inhibitor. The quercetin and amoxicillin were pre-incubated with the enzyme at 37 °C for 5 min before substrate addition. Reaction samples were injected at various times to Waters Bio-Sil C18 HL 90-5 s reverse phase column. Time-course assays were carried out using methanol/acetic acid (100:1) as stopping reagent. The analyzes of the remaining substrate were determined by reverse-phase HPLC using acetonitrile/ammonium acetate as a mobile phase [26].

### Transmission electron microscopy (TEM)

Cellular damage of bacteria was examined using TEM. Amoxicillin and quercetin that dramatically decreased the MICs against ARSE 5038 were chosen for electron microscopy study when used singly and in combination. The subculture of this strain was prepared to examine by TEM following Eumkeb et al. [19]. Bacterial cells treated with 8 μg/mL amoxicillin, 128 μg/mL, quercetin (½ MIC) and 3 μg/mL amoxicillin plus 48 μg/mL quercetin (¾ FIC) were harvested after log phase of incubation and fixed in 2.5 % glutaraldehyde in 0.1 M PBS at 4 °C for 2 h. The cells were washed, postfixed for 2 h in 1 % osmium tetroxide in 0.1 M PBS (pH 7.2). After washing twice with PBS, the cells were dehydrated, infiltrated and embedded in Spur’s resin. Ultrathin sections were cut, mounted on bare copper grids. Finally, specimens were counterstained with 2 % (w/v) uranyl acetate solution for 15 min and then with 0.25 % (w/v) lead citrate solution for 15 min and examined with a TECHNAI G^2^ electron microscope (FEI, USA) operated at 120 kV.

### Immunofluorescence staining and confocal microscopy

Disruption of peptidoglycan and DNA leakage after exposure to quercetin plus amoxicillin was carried out by the immunofluorescence and visualized under a confocal laser scanning microscope following the method of Teethaisong et al. [27]. In brief, after the FIC index was elucidated from checkerboard, the half-MICs value of quercetin or amoxicillin alone and the ¾ FIC of this combination that gave synergistic FIC indices were selected against ARSE 5038. The cell grown without an antibacterial agent was used as control [28].

### Fourier Transform-Infrared (FT-IR) microspectroscopy measurement

#### Chemicals, bacterial strain and growth conditions

To evaluate the effect of quercetin either alone or in combination with amoxicillin on ARSE 5038 cells using FT-IR measurement, the previous method of Eumkeb et al. was followed [11]. Shortly, after the FIC index was obtained from checkerboard, the half-MICs value of quercetin or amoxicillin alone and the ¾ FIC of this combination that gave synergistic FIC indices were selected against ARSE 5038 to perform the FT-IR investigation. These cells were incubated at 37 °C in shaking water bath for 4 h. The cell pellets were centrifuged at 3000 *xg* for 10 min and washed twice with saline [11]. These cells were then washed twice with Milli-Q water. A small portion of the pellet was then deposited on Mirr IR low e-microscope slides (Kevey slide) to use as a substrate for FT-IR microscope analysis. These cells were then desiccated under vacuum about 20 min and stored in desiccators to form films suitable for analysis [29].

### Data preprocessing and analysis

To achieve high S/N ratios, 64 scans coadded were collected for each measurement in the wavenumber between 4000 and 400 cm^−1^ resolution of 6 cm^−1^. Spectra were recorded in reflection mode on a Bruker IR spectrometer (tensor 27) coupled to an IR microscope (Hyperion 2000) with 36× magnification. The OPUS 6.5 software (Bruker Optics, German) and the Unscrambler 9.7 software (Camo, Norway) was used to calculate the signal intensity of second derivatives and the band areas over the band baseline, give information about the concentration of the functional groups responsible for the corresponding band, was calculated and compared [12, 30].

To analyze the effect of variation in the composition and distribution of the biochemical components in bacterial cells during cell culture, the data were analyzed using PCA. All data analysis was carried out in the spectral range from 3000-2800 cm^−1^ and 1800-850 cm^−1^, which cover the mixed region of lipid, protein, polysaccharide and the “true” fingerprint region [9, 31].

### Statistical analysis

All experiments were carried out in triplicate, and average values with a standard error of mean (Mean ± SEM) were indicated. Significant differences between treated groups were compared using ANOVA. A *p*-value < 0.01 of Scheffe’s posthoc test, denoted the presence of a statistically significant difference.

## Results

### MICs and checkerboard determinations

The MICs of testing penicillin, nisin, and flavonols (quercetin and kaempferol; Fig. 1) against all *S. epidermidis* DMST strains are shown in Table 1. The results revealed that MICs of amoxicillin, penicillin, nisin, quercetin and kaempferol against all of these *S. epidermidis* strains were 16, 200, 256, 256-384, and >1024 μg/mL respectively. These results provide evidence that these strains are resistant to amoxicillin and penicillin. Quercetin exhibited little inhibitory effect against these strains. The FIC indices of amoxicillin plus quercetin and penicillin plus kaempferol against all of these strains were 0.50 and <0.38 respectively. So, these combinations showed synergistic activity against all of these *S. epidermidis* strains as above mention (Checkerboard determination) [17, 32]. *S. aureus* ATCC 29213 was used as positive controls.

**Table 1:** MICs, FIC, and FIC index of amoxicillin, penicillin, quercetin, kaempferol against *S. epidermidis*

Strains	MIC (μg/mL)					FIC (μg/mL) = FIC index	
	Amx	Pen	Nis	Que	Kae	Amx + Que	Pen + Kae
*S. epidermidis* DMST 5038	16^*R*^	200^*R*^	256	256	>1024	4 + 64 = 0.5^*SI*^	64 + 64 = < 0.38^*SI*^
*S. epidermidis* DMST 5023	16^*R*^	200^*R*^	256	256	> 1024	4 + 64 = 0.5^*SI*^	64 + 64 = < 0.38^*SI*^
*S. epidermidis* DMST 5868	16^*R*^	200^*R*^	256	384	> 1024	4 + 96 = 0.5^*SI*^	64 + 64 = < 0.38^*SI*^
*S. epidermidis* DMST 4248	16^*R*^	200^*R*^	256	384	> 1024	4 + 96 = 0.5^*SI*^	64 + 64 = < 0.38^*SI*^
*S. aureus* ATCC 29213^a^	0.5^*S*^	0.5^*S*^	1	N/D	N/D	N/D	N/D

Each compound was measured three times

*MIC* Minimum inhibitory concentrations, *FIC* Fractional inhibitory concentration, *Amx* amoxicillin, *Pen* penicillin, *Nis* nisin, *Que* quercetin, *Kae* kaempferol

^*S*^ = Susceptible; ^*R*^ = resistant; ^*SI*^ = Synergistic interaction; *N/D* not determined

^a^ *S. aureus* ATCC 29213 was used as a positive control

### Killing curve determinations

**Fig. 2:**
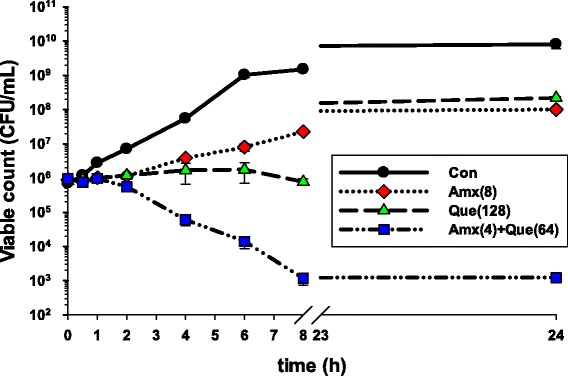
The effect of amoxicillin, quercetin on the viable counts of ARSE 5038. Con = control (drugs free); Amx (8) = 8 μg/mL Amoxicillin; Que (128) = 128 μg/mL Quercetin; Amx (4) + Que (64) = 4 μg/mL Amoxicillin plus Quercetin 64 μg/mL. The values plotted are the means of 4 observations, and the vertical bars indicate the standard errors of the means

The effect of amoxicillin and quercetin either alone or in combination on viable counts of ARSE 5038 is revealed in Fig. 2. The viable count of the cells treated with quercetin 128 μg/mL alone displayed slightly lower than amoxicillin 8 μg/mL alone between 2 and 8 h. However, these cells were gradually recovered after eight throughout 24 h. Obviously, the combination of amoxicillin at 4 μg/mL plus quercetin at 64 μg/mL dramatically decreased the cells to 1×10^3^ CFU/mL after 8 h and maintained the cells count at this level throughout 24 h. The synergistic activity of this combination has been confirmed by ≥ 2 log10 CFU/mL reduction of these treated cells compared to amoxicillin treat alone [33].

### Cytoplasmic membrane (CM) permeability assay

**Fig. 3:**
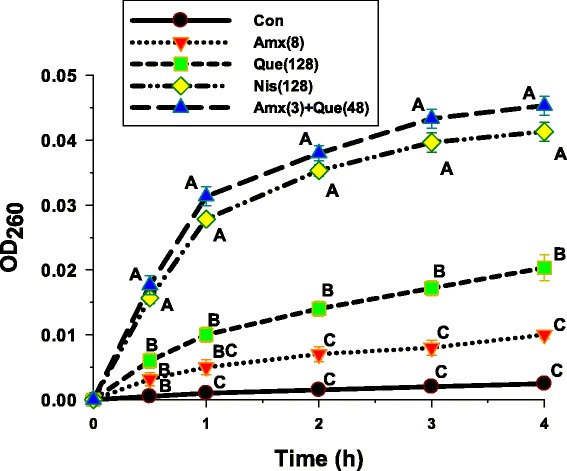
The presence of 260 nm absorbing materials of ARSE 5038 treated with quercetin, amoxicillin. Con = control (drugs free), Amx (8) = 8 μg/mL Amoxicillin; Que (128) = 128 μg/mL Quercetin; Nis (128) = 128 μg/mL Nisin; Amx (3) + Que (48) = 3 μg/mL Amoxicillin plus 48 μg/mL Quercetin. Nisin at 128 μg/mL was used as positive control, and untreated cells were used as negative control. The mean ± SEM for three replicates are illustrated. Means sharing the same superscript are not significantly different from each other (Scheffe’s test, *p* < 0.01)

The cytoplasmic membrane permeability was measured by UV-absorbing release materials as demonstrated in Fig. 3. After treatment ARSE 5038 cells with 8 μg/mL amoxicillin, 128 μg/mL nisin, 128 μg/mL quercetin and 3 μg/mL amoxicillin plus 48 μg/mL, quercetin alone could induce the release of 260 nm absorbing material, which we interpret to be mostly DNA, RNA, and metabolites significantly higher than control and amoxicillin within 1 and 2 h respectively (*p* < 0.01). The absorbance values of amoxicillin plus quercetin and nisin treated groups were significantly higher than those of amoxicillin, quercetin treated alone and the negative control from 0.5 h and throughout 4 h (*p* < 0.01). These results imply that quercetin alone and in combination with amoxicillin increased cytoplasmic membrane permeability of this strain [20, 21].

### Enzyme assay

**Fig. 4:**
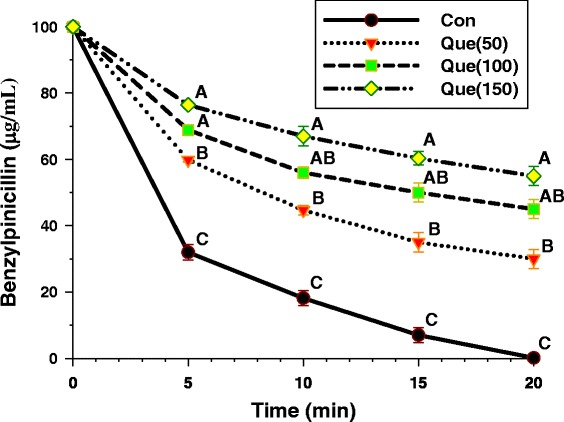
The inhibitory activity of quercetin against β-lactamase in hydrolyzing benzylpenicillin. β-lactamase used from *E. cloacae*; Con = control (no testing agent); Que (50) = Quercetin 50 μg/mL. The graph shows the remaining benzylpenicillin at the same time. Means sharing the same superscript are not significantly different from each other (Scheffe’s test, *p* < 0.01)

The capability of quercetin to inhibit the activity of β-lactamase type IV from *E. cloacae* is shown in Fig. 4. The result displayed that benzylpenicillin treated with quercetin was significantly higher than control starting from 5 min (*p* < 0.01). The benzylpenicillin remainder was significantly increased by a rise in a concentration-dependent manner. These results suggest that one activity of quercetin against ARSE may involve in β-lactamase inhibition [19].

### Transmission electron microscopy (TEM)

**Fig. 5:**
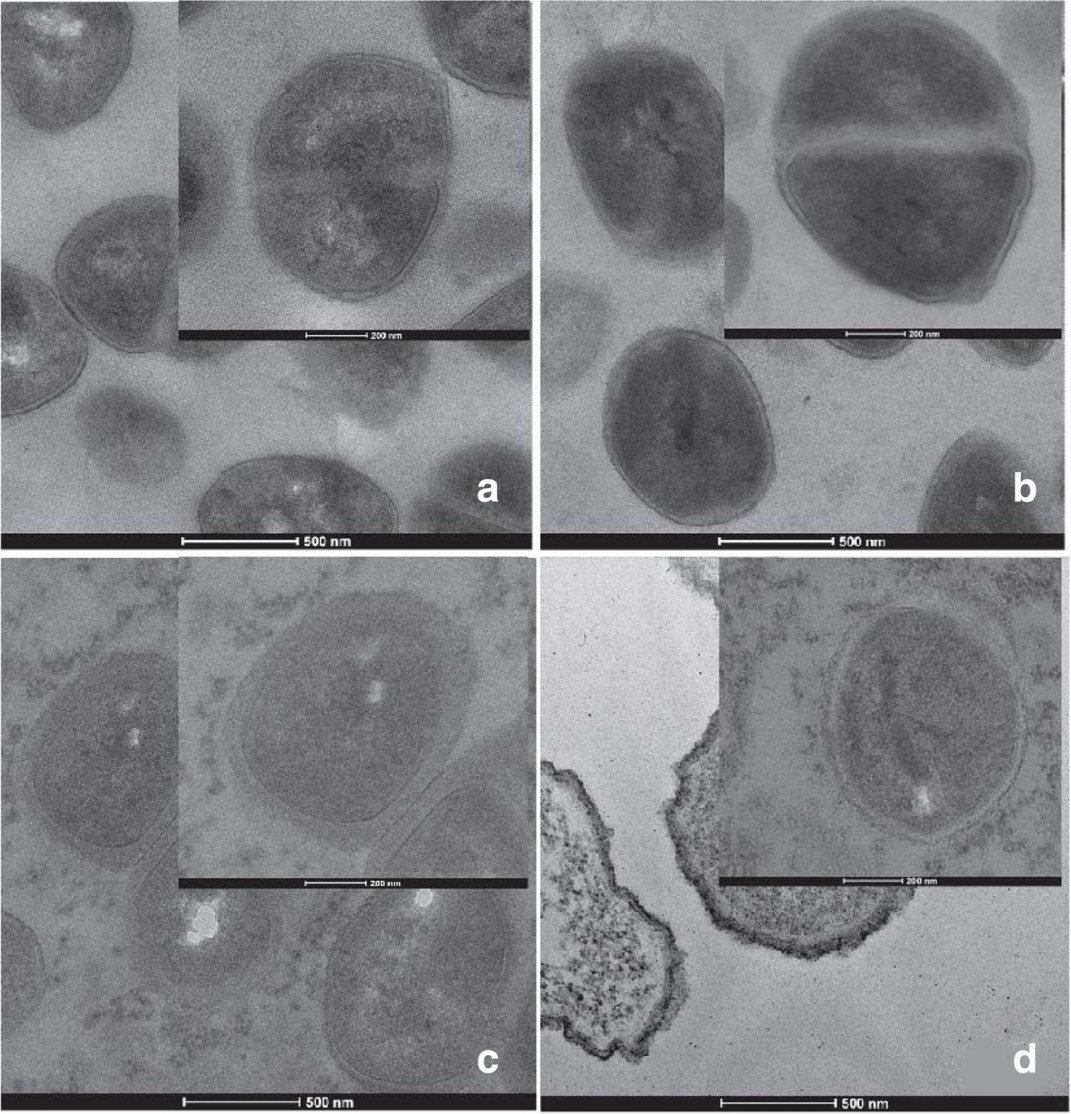
Ultrathin sections of log phase ARSE 5038 grown in CAMHB containing: (**a**) control (drug-free); (**b**) Amoxicillin (8 μg/mL); (**c**) Quercetin (128 μg/mL); (**d**) Amoxicillin (3 μg/mL) plus Quercetin (48 μg/mL) (a, b, d, original magnification, 19500×; c, 17000×, bar, 500 nm; ***Inset***: a, b 38000×; bar, 200 nm, c, d, 34000×; bar, 200 nm)

**Fig. 6:**
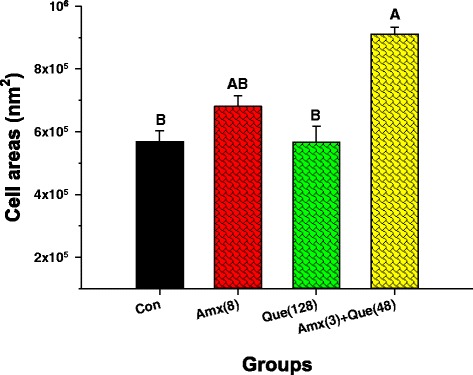
The effect of amoxicillin, quercetin on an average cross-section of ARSE 5038 cell areas. Con = control (drugs free); Amx (8) = 8 μg/mL Amoxicillin, Que (128) = 128 μg/mL Quercetin, Amx (3) + Que (48) = 3 μg/mL Amoxicillin plus 48 μg/mL Quercetin. The mean ± SEM for three replicates are illustrated. Means sharing the same superscript are not significantly different from each other (Scheffe’s test, *p* < 0.01)

Electron micrographs of log phase of ARSE 5038 cells in the presence of amoxicillin, quercetin either alone or in combination are shown in Fig. 5a to d. The peptidoglycan and cytoplasmic membrane can be distinguished from the control group. The morphology of the cells looked normal appearance (Fig. 5a). The ARSE cells treated with amoxicillin are revealed in Fig. 5b. This result displayed a minority of detrimental peptidoglycan and few cytoplasmic membrane hurt. The average cross-sectional cell areas of these cells were bigger than control, but not a significant difference (*p* < 0.01) (Fig. 6). Whereas, the micrograph of these cells after exposure to quercetin alone is shown in Fig. 5c. The result exhibited that some of these cells revealed peptidoglycan and cytoplasmic membrane damage. The average cell areas of these cells were approximately the same as a control (*p* < 0.01) (Fig. 6). Figure 5d reveals this amoxicillin plus quercetin-treated cells. These cells demonstrated that a majority of these cells exhibited marked morphological damage, noticeable peptidoglycan and cytoplasmic membrane damage, electron transparent areas devoid of the ribosome. Obviously, these average cell areas were significantly bigger than the control (*p* < 0.01) (Fig. 6).

**Fig. 7:**
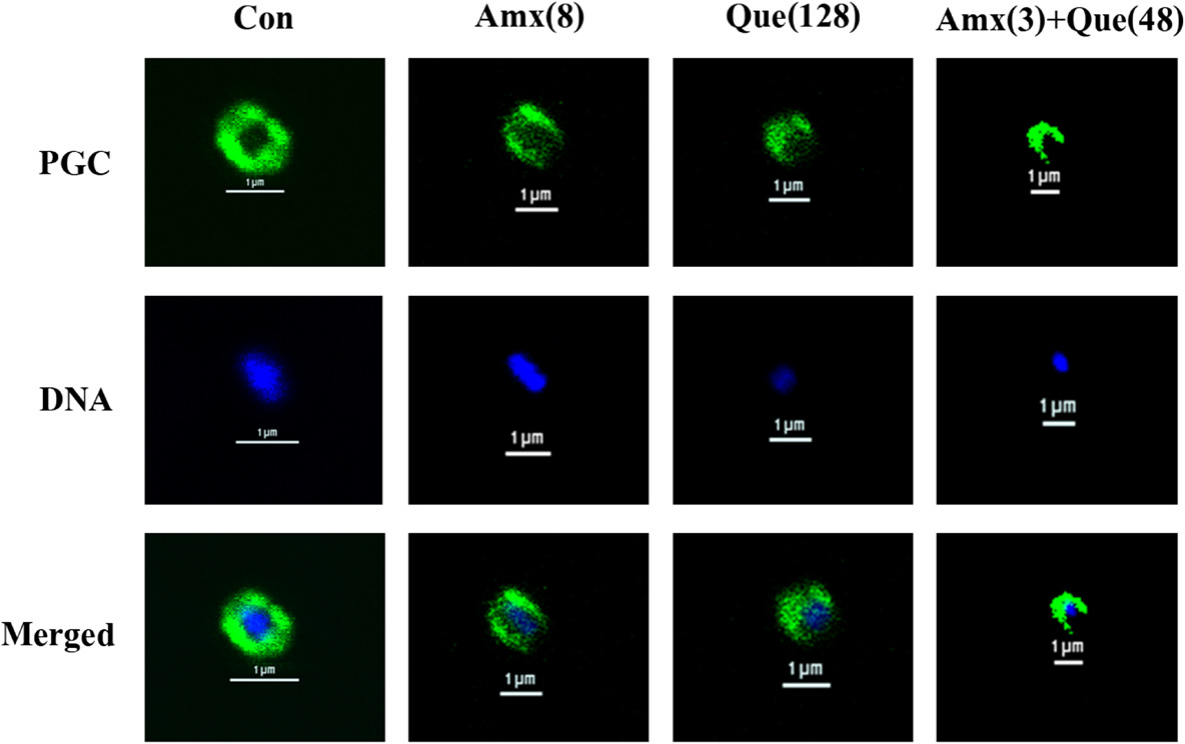
Schematic representation of the results of immunofluorescence and a confocal laser scanning microscope; Samples of ARSE after treatment for 4 h with Amoxicillin, Quercetin, either alone or in combination. Con = control (drugs free); Amx (8) = 8 μg/mL Amoxicillin; Que (128) = 128 μg/mL Quercetin; Amx (3) + Que (48) = 3 μg/mL Amoxicillin plus 48 μg/mL Quercetin. The cells were stained for DNA with DAPI (blue) and labelled for peptidoglycan (PGC) (green) using respective antibodies. DNA in all groups was localized in the central of the cell and surrounded by a peptidoglycan layer (merged images). Scale bar = 1 μm

### Immunofluorescence staining and confocal microscopy

The peptidoglycan and DNA-labeled ARSE 5038 clearly showed intact coccus-shaped and no damage was observed in control cell by confocal laser scanning images (Fig. 7). These cells treated with amoxicillin and quercetin alone revealed a little damage to peptidoglycan and DNA leakage, although quercetin treated alone exhibited more peptidoglycan damage and DNA leakage than ampicillin alone. The combination of these agents caused considerable peptidoglycan damage and DNA leakage compared to controls. The merger of peptidoglycan and DNA images are also shown. These results are in substantial correspondence with TEM outcomes and support a preliminary mechanism of action of this combination is probably inhibiting peptidoglycan synthesis.

### FT-IR spectroscopy measurement

**Fig. 8:**
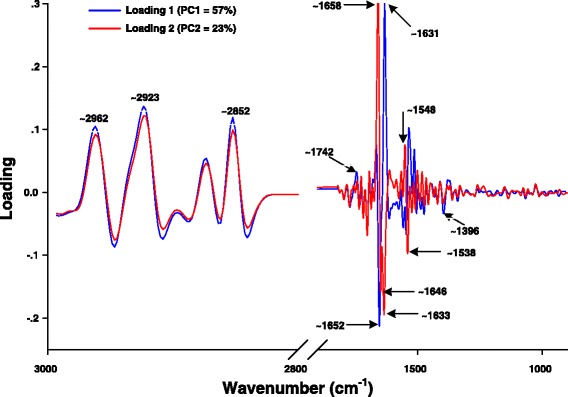
The loading plot of the 1^st^ and 2^nd^ principal components from PCA of ARSE 5038. FT-IR spectra of control (drugs free), 8 μg/mL Amoxicillin; 128 μg/mL Quercetin; and 3 μg/mL Amoxicillin plus 48 μg/mL Quercetin demonstrated wavenumber at 3000-2800 cm^−1^ and 1742-1396 cm^−1^

**Fig. 9:**
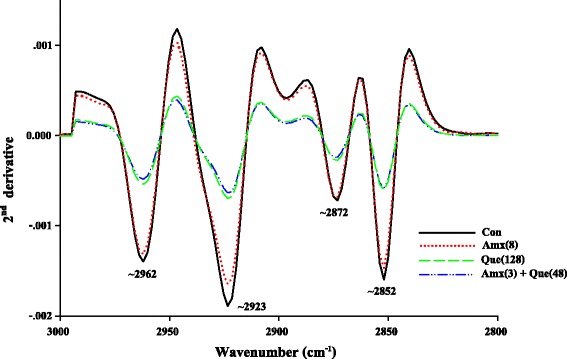
Representatives 2^nd^ derivative transformation spectra (3000–2800 cm^−1^) of ARSE 5038 treated with Quercetin, Amoxicillin. Con = control (drugs free), Amx (8) = 8 μg/mL Amoxicillin; Que (128) = 128 μg/mL Quercetin; Amx (3) + Que (48) = 3 μg/mL Amoxicillin plus 48 μg/mL Quercetin

**Fig. 10:**
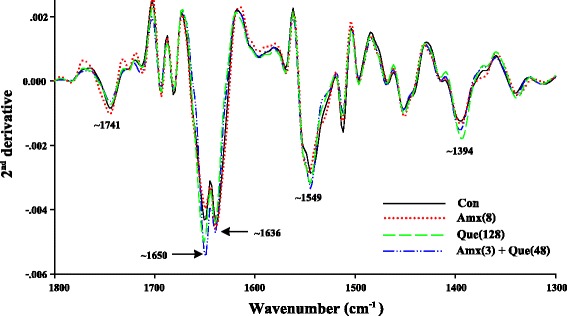
Representative 2^nd^ derivative transformation spectra (1800–1300 cm^−1^) of ARSE 5038 treated with Quercetin, Amoxicillin. Con = control (drugs free), Amx (8) = 8 μg/mL Amoxicillin; Que (128) = 128 μg/mL Quercetin; Amx (3) + Que (48) = 3 μg/mL Amoxicillin plus 48 μg/mL Quercetin

The ARSE 5038 strain was grown in CAMHB medium in the presence of 8 μg/mL amoxicillin (½ MIC), 128 μg/mL quercetin (½ MIC) and 3 μg/mL amoxicillin plus 48 μg/mL quercetin (¾ FIC) and examined by FT-IR microspectroscopy. The loading plots are presented in Fig. 8. The 1^st^ loading of amoxicillin and control groups indicate that distinct regions at 3000-2800 cm^−1^ (~2962, ~2923, ~2852 cm^−1^) and ~1742 cm^−1^ correspond to stretching mode of CH_2_ and CH_3_ of fatty acids of the various membrane amphiphiles and ester band respectively [31]. For this reason, the signal intensity and area of the peaks of these fatty acid regions of quercetin either alone or in combination with amoxicillin clearly showed lower than those of amoxicillin and control (Fig. 9). Also, the 2^nd^ loading displays 3 region coefficients at ~1646, ~1633 and ~1548 cm^−1^ (Fig. 8). These regions relate to average bands that are shown in Fig. 10. These cells after treatment with quercetin either alone or in combination with amoxicillin exhibited higher signal intensity and band areas at ~1650, ~1636 and ~1549 cm^−1^ which are attributed to an absorption peak of secondary structure of protein amide I (alpha-helix and beta-sheet) and amide II than amoxicillin and control groups [31].

**Fig. 11:**
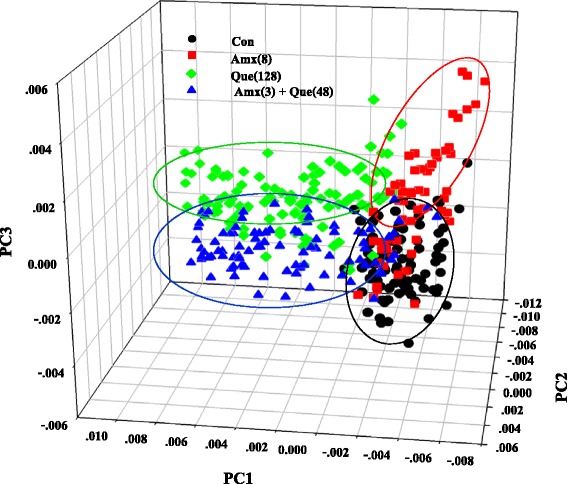
PCA results constructed using spectra of ARSE 5038 treated with Quercetin, Amoxicillin. Con = control (drugs free), Amx (8) = 8 μg/mL Amoxicillin, Que (128) = 128 μg/mL Quercetin, Amx (3) + Que (48) = 3 μg/mL Amoxicillin plus 48 μg/mL Quercetin. PCA analysis (Ward’s algorithm method) was constructed by using the second derivative, vector normalized spectra, over the spectral ranges from 3000-2800 cm^−1^ and 1750-900 cm^−1^

The principle component analysis (PCA) can be explained by the primary source of variation in the fingerprint region to differentiate and classify bimolecular of bacterial cells after treatment with quercetin, amoxicillin either alone or in combination [34]. The 3-dimensional PCA clustering results from FT-IR spectral data of ARSE 5038 after treatment with amoxicillin, quercetin either alone or in combination is displayed in Fig. 11. The biomolecular fingerprint clusters between quercetin, amoxicillin either alone or in combination and control groups were clearly differentiated. This differentiation and classification most likely imply compositional and structural impacts of the bactericidal effect of quercetin either alone or in combination with amoxicillin on amide I components of proteins, fatty acids, polysaccharides and nucleic acids [35]. The 1^st^ principal component (PC1) characterizes the maximum percentage of bacterial spectral variation follow by the PC2 [36].

The loading from PC1 of ARSE 5038 cells after treatment with quercetin either alone or in combination with amoxicillin were accounted for 80 % of the total variability (PC1 57 and PC2 23 %) and case of treating group loading PC2 was accounted for 66 % of the total variability (PC2 57 and PC3 9 %).

## Discussion

Flavonoids have inhibitory activity against a variety of bacteria. The structure of flavonoids was isolated and identified. Many researchers described that flavonoids possessed antibacterial activity, including quercetin, and various quercetin glycosides [37].

The MIC results revealed that this testing *S. epidermidis* strains were resistant to amoxicillin and penicillin alone because of the standard value of the sensitivity of both amoxicillin and penicillin against these strains are 0.5 μg/mL [15]. As well as quercetin, and kaempferol demonstrated little bacteriostatic effect against these strains. Likewise, these results are in substantial agreement with those of Hirai et al. [38] that the relatively high concentration of quercetin at 50 μM showed a weak inhibitory effect against *S. aureus* and *S. epidermidis* at 3–9 h of incubation. Besides, 3-(quercetin-8-yl)-2,3-epoxyflavanone, new quercetin-derived oxidation products, at 100 μg/disk showed antibacterial activity against MRSA and *H. pylori* strains at 15–16 and 12–13 mm respectively and increased susceptibility of MRSA to 10 μg/mL oxacillin by increase inhibition zone from 1 to 18 mm [39]. Also, quercetin at 100–1000 μg/mL displayed inhibition zone 2.4–3.8 mm against *S. aureus* ATCC 29213 and 25923 [40]. As well as, the MICs of quercetin and amoxicillin against penicillin-resistant *S. aureus* strains were > 400 and 250 μg/mL, respectively [19]. The checkerboard determination revealed synergistic effects of amoxicillin plus quercetin and penicillin plus kaempferol against all of tested *S. epidermidis* strains with FIC indexes at 0.50 and < 0.38 respectively [17]. The synergy effect of amoxicillin and quercetin against ARSE 5038 has been confirmed by killing curve due to these cells have been reduced ≥ 2 log10 CFU/mL [33]. These results are in substantial correspondence with those of Eumkeb et al. that quercetin plus amoxicillin exhibited synergistic activity against penicillin-resistant *S. aureus* strains at FIC indices < 0.05 [19]. In the same way, previous studies reported that a synergistic effect of quercetin and oxacillin against vancomycin-intermediate *S. aureus* displayed the lowest FIC index value of 0.0417 [41]. Apart from this, the antibacterial activity of quercetin plus ampicillin or vancomycin against the sensitive MRSA strain were significantly increased compared to control (no any testing agent) (*p* < 0.01) [38]. As one might expect, the quercetin and cobalt (II), cadmium (II), or mercury (II) complexes at 100 μg/disc showed significant activity as bactericide against *S. aureus* at 10, 15 and 20 mm inhibition zone respectively, whereas sodium penicillin at 100 μg/disc showed inhibition zone at 17 mm [42].

Naturally, the isolated *Staphylococcus epidermidis* strains can produce β-lactamase [22], and the result from enzyme assay found that quercetin had an inhibitory activity against β-lactamase type IV from *E. cloacae.* Hence, these results provide evidence that one mode of action of quercetin against ARSE may involve in β-lactamase inhibition and may cause the resistance of bacterial strains to its sensitivity to an antibiotic [24]. Clearly, these results seem consistent with previous findings that galangin and kaempferide, both are flavonols, showed marked inhibitory activity against penicillinase (β-lactamase) type IV from *E. cloacae* [19, 24].

The CM permeability revealed that quercetin either alone or in combination with amoxicillin increased cytoplasmic membrane permeability of this strain. These results are in substantial agreement with previous findings that luteolin either alone or combined with amoxicillin and apigenin alone and in combination with ceftazidime increased CM permeability of amoxicillin-resistant *E. coli* and ceftazidime-resistant *E. cloacae* respectively [11, 43]. The increase in CM permeability may be one of the synergistic action of this combination against ARSE strain. This result could be explained that the formation of pores in the plasma membrane might be disrupted [21].

TEM results of amoxicillin plus quercetin-treated cells demonstrated that these cells exhibited marked morphological damage, thin peptidoglycan, and cytoplasmic membrane damage, electron transparent areas devoid of the ribosome and average cell areas significantly bigger than the control (*p* < 0.01). These results seem consistent with previous findings that the combination of ceftazidime plus galangin caused damage to the ultrastructures of the cells, affected the integrity of the cell walls and led to an increase in cell size of ceftazidime-resistant *S. aureus* [19]. Confocal microscopic images have verified the TEM results that the peptidoglycan of this combination treated cells was obviously damaged. These findings can be explained by assuming that quercetin may insert synergistic action with amoxicillin to inhibit peptidoglycan synthesis leads to marked morphological damage and delay cell division.

In general, previous findings revealed that the bactericidal effect of chlorine caused changes in the second derivative ATR spectra because of alteration in bacterial ester functional groups of lipids, structural proteins, and injured bacterial cells [9]. Our FT-IR results exhibited that ARSE 5038 cells treated with quercetin either alone or in combination with amoxicillin were decreased in fatty acid, but increased in protein amide I and amide II in bacterial cells compared to control. These results lend us to believe that quercetin alone and combined with amoxicillin may affect the content of fatty acid chains on the different membrane amphiphiles results in cytoplasmic membrane damage and an increase in cytoplasmic membrane permeabilisation. Whereas, this combination may form a complex with β-lactamase, transpeptidase, and other proteins lead to stacked- and accumulated protein in bacterial cells.

These findings provide evidence that quercetin alone has not only rather weak activity against ARSE, but also possess the ability to reverse the resistance to its first sensitivity to an antibiotic. This synergistic activity of quercetin plus amoxicillin may involve four modes of actions of this flavonol: (1) Insert action to inhibit peptidoglycan synthesis, resulting in morphological damage. (2) Inhibit β-lactamases activity. (3) Increase CM permeability and (4) decrease fatty acid, but increase protein amide I and amide II on bacterial cells. These mechanisms of actions are practically in substantial correspondence with previous findings that mode of synergistic actions of galangin, a flavonol, plus amoxicillin against ceftazidime-resistant *S. aureus* may act via three mechanisms that that inhibit protein synthesis, interact with penicillinase and cause cytoplasmic membrane damage [19].

The currently β-lactamase inhibitors could be broken by β-lactamase via a mechanism similar to the β-lactam antibiotics and could induce β-lactamase production [44]. The structure of quercetin is completely different from β-lactamase inhibitors. So, this flavonol is unlikely to induce β-lactamase production. Quercetin may be developed to combine with amoxicillin as the new combination of phytopharmaceuticals for the treatment of ARSE infection that cannot be treated with amoxicillin alone. The previous study reported that the bioavailability of quercetin in healthy individuals was relatively poor as evidenced by limited increases in its circulating concentrations after oral ingestion of 100 mg quercetin glucoside [45]. Although, Murota et al. found that enzymatic a-oligoglucosylation to the sugar moiety was effective for enhancing the bioavailability of quercetin glucosides in humans [46].

## Conclusions

In summary, our study provides evidence that quercetin has the extraordinary potential to reverse bacterial resistance to originate traditional drug susceptibility of it. This finding is the first report of the mechanism of synergistic action of flavonol plus penicillins combination against amoxicillin-resistant *S. epidermidis* using FT-IR. Four modes of actions would be implied that this combination inhibits peptidoglycan synthesis, inhibit β-lactamases activity, increase CM permeability, and decrease fatty acid, but increase protein amide I and amide II on bacterial cells. Naturally, quercetin has restricted, limited toxicity. So, this flavonol proposes the high potential to develop a useful of novel adjunct phytopharmaceutical to amoxicillin for the treatment of ARSE. Future studies should be investigated that its bioavailability is effective to be used as antibiotics in humans. Also, the synergistic effect on blood and tissue would be evaluated and achieved.

## Abbreviations

ARSE 5038, amoxicillin-resistant *Staphylococcus epidermidis* DMST 5038; ARSE, amoxicillin-resistant *Staphylococcus epidermidis;* CAMHB, cation-adjusted Meuller-Hinton broth; CM, cytoplasmic membrane; DMSO, dimethyl sulfoxide; FIC, fraction inhibitory concentration; FICI, fraction inhibitory concentration index; FT-IR, fourier transform-infrared; MHA, Meuller-Hinton agar; MHB, Meuller-Hinton broth; MIC, minimum inhibitory concentration; TEM, transmission electron microscopy
